# 

**Published:** 1860

**Authors:** 


**Payments.**

J. A. Johnson, $3; A. H. Shi, $10; N. H. Baker, $6; Boland, $1; Jas. N. Coe, $3; J. C. Avery, $3; J. G. W. Brown, $3; J. W. Leak, $1; W. P. Hardin, $3; Joseph W. Clift, $3; A. Willingham, $6.50; R. A. Fontaine, $3; B. F. C. Bonner, $3; J. P. Taylor, $3; J. E. G. Terrell, $3; Jas. Thweatt, $3; G. W. Powell, $6; N. T. Bridges, $2; P. M. Tidwell, $9; J. G. Cantrell, $3; W. T. Landrum, $3; Jno. Stone, $3; A. V. Mann, $3; M. V. D'Aguiar, $3; W. H. Stringfellow, $10; C. F. Robinson, $5.

